# Disclosure of medical errors: physicians’ knowledge, attitudes and practices (KAP) in an oncology center

**DOI:** 10.1186/s12910-020-00513-2

**Published:** 2020-08-20

**Authors:** Razan Mansour, Khawlah Ammar, Amal Al-Tabba, Thalia Arawi, Asem Mansour, Maysa Al-Hussaini

**Affiliations:** 1 University of Jordan, School of Medicine, Amman, Jordan; 2 Office of Scientific Affair and Research, King Hussein Cancer Center, Amman, Jordan; 3 Office of Human Research Protection Program, King Hussein Cancer Center, Amman, Jordan; 4 Salim EL Hoss Bioethics and Professionalism Program, American University of Beirut Medical Center, Beirut, Lebanon; 5 Chair, Institutional Review Board Office, King Hussein Cancer Center, Amman, Jordan

**Keywords:** Medical error, Disclosure, Law, Training

## Abstract

**Background:**

Between the need for transparency in healthcare, widely promoted by patient’s safety campaigns, and the fear of negative consequences and malpractice threats, physicians face challenging decisions on whether or not disclosing medical errors to patients and families is a valid option.

We aim to assess the knowledge, attitudes and practices (KAP) of physicians in our center regarding medical error disclosure.

**Methods:**

This is a cross-sectional self-administered questionnaire study. The questionnaire was piloted and no major modifications were made.

A day-long training workshop consisting of didactic lectures, short and long case scenarios with role playing and feedback from the instructors, were conducted. Physicians who attended these training workshops were invited to complete the questionnaire at the end of the training, and physicians who did not attend any training were sent a copy of the questionnaire to their offices to complete. To assure anonymity and transparency of responses, we did not query names or departments.

Descriptive statistics were used to present demographics and KAP. The differences between response\s of physicians who received the training and those who did not were analyzed with t-test and descriptive statistics. The 0.05 level of significance was used as a cutoff measure for statistical significance.

**Results:**

Eighty-eight physicians completed the questionnaire (55 attended training (62.50%), and 33 did not (37.50%)). Sixty Five percent of physicians were males and the mean number of years of experience was 16.5 years. Eighty-Seven percent (*n* = 73) of physicians were more likely to report major harm, compared to minor harm or no harm. Physicians who attended the workshop were more knowledgeable of articles of Jordan’s Law on Medical and Health Liability (66.7% vs 45.5%, *p*-value = 0.017) and the Law was more likely to affect their decision on error disclosure (61.8% vs 36.4%, *p*-value = 0.024).

**Conclusion:**

Formal training workshops on disclosing medical errors have the power to positively influence physicians’ KAP toward disclosing medical errors to patients and possibly promoting a culture of transparency in the health care system.

## Background

Medical error disclosure is defined as “communication between a health care provider and a patient, family members, or a patient’s proxy that acknowledges the occurrence of an error, discusses what happened, and describes the link between the error and outcomes in a manner that is meaningful to the patient” [1]. Despite the increasing efforts health care institutions and health care workers make to prevent medical errors and adverse events [2], medical errors are still inevitable. In the United States, more than one million medical errors occur annually, and the mortality from medical errors is estimated to be about 98,000 deaths/year [3]. In Jordan, a study found that 28% of all hospital admissions in Jordan were affected by medical errors, with medication errors as the most common cause, followed by the wrong medical diagnosis and health care associated infections. Wrong diagnosis, infections, bedsores and falls were reported by 21.3, 21.3, 16 and 8% of participants, respectively [4]. Other errors reported in the study were errors of patient identification, transfusion errors, and medical errors that lead to patients’ death.

Disclosure of medical errors is recommended and governed by ethical and professional guidelines and legislations [5, 6], and the acknowledgement of medical errors, even if those errors classify as minor or insignificant, is desired by the overwhelming majority of patients [7, 8]. Furthermore, patients were more inclined to consider the litigation of medical errors if their physician did not disclose them [9], which further reinforces the importance of the physician’s knowledge on how to properly disclose medical errors to their patients [10, 11]. Possible outcomes also include when and where to disclose the errors.

Interestingly, the prevalence of disclosure of medical errors remains fluctuant, as studies found that 50 to 96% of errors in the US are underreported by physicians [12, 13]. Nevertheless, physicians still acknowledge disclosure of medical errors as a fundamental part of patients’ care [14–17]. While trust is the cement that holds the physician-patient relationship, concealing errors fractures trust and leads to distrust in the medical profession writ large. To further emphasize the importance of disclosing errors to patients, there is a need to better understand the elements that interfere with error disclosure [18]. Challenges and barriers of disclosing medical errors are not limited only to the fear of disclosing the event to the patients, but also include the repercussions of discussion with peers such as blame, embarrassment or even loss of reputation [19, 20]. It also includes proper reporting to the institution, and the possibility of legal liability [14, 21–23].

Given the importance of a physician’s knowledge and willingness to report medical errors in improving healthcare standards and quality, King Hussein Cancer Center (KHCC) in collaboration with the Salim El-Hoss Bioethics and Professionalism Program (SHBPP) at the American University of Beirut Faculty of Medicine and Medical Center (AUBMC) conducted several workshops, which aimed to coach physicians at KHCC on how, when and where to effectively disclose medical errors to patients and their families, especially in view of the recently enacted “Medical and Health Liability Law” (MHLL) in Jordan which was published by the government of Jordan and took effect on the 31st of August, 2018 [24]. Article 5 of the MHLL illustrates that physicians are required to perform services subject to the requirements of the morals, accuracy, and loyalty of the profession and they must also be in line with the established scientific standards. According to what is stated by law, a medical error occurs when physicians are involved in an act or omission, which does not correspond to the standard professional rules resulting in harm to the patient. The aim of the law is to better the government and regulation of the relationship between physicians and patients, especially when a medical error is suspected.

The King Hussein Cancer Center is a standalone, renowned and comprehensive cancer center in the Middle East, which treats both adult and pediatric patients. It provides a state of the art cancer care for both the citizens of Jordan and many neighboring countries. The center treats over 6000 new cases and its outpatient clinics have almost 300,000 encounters annually [25].

Although disclosure of medical errors remains consequential in all medical fields, medical errors have specialty-sensitive dimensions [26], one example would be when it comes to cancer patients. Cancer care can be complex and integrative, as the use of many medications, the tendency for cancer patients to be enrolled in clinical trials, the long follow up time frame, and the involvement of multidisciplinary teams can make it especially challenging for physicians and may add to the risk of medical errors and to the difficulty of disclosing them to patients [27]. Moreover, given the vulnerability of this group, disclosing medical errors to pediatric cancer patients is of a particular challenge, as they may especially face diagnostic delays given the nature of disease in pediatric population [28], chemotherapy infusion errors [29], and outpatient adverse events [30]. Given the complexity of error disclosure in cancer patients, ethical professional practice in KHCC is driven by its six core values of cultural and ethical sensitivity, excellence, compassion, teamwork, innovation, and person-centered care. Ethical practice is reflected in the various medical and administrative functions of the institution, from decision making at the Hospital Ethics Committee [31] for dealing with ethical issues that arises when delivering the standard of care procedures, to the review of human subject research at the Institutional Review Board [32] to ensure only ethically sound research is conducted at the center.

The aim of this cross-sectional study is to assess the knowledge, attitudes and practices (KAP) of physicians at KHCC towards error disclosure in addition to the effect of the formal training workshops on physicians’ KAP.

## Methods

This is a cross sectional self-administered questionnaire study. The questionnaire was based mainly on the work of Kaldijian et al. (2007) [33]. The research team obtained permission to use the questionnaire from the study’s first author then made some modifications and omissions to adapt to the KHCC culture (Supplementary File-1). The questionnaire was administered in English, as it is the formal language used during training and the routine medical documentation. A 5-point Likert scale was used ranging from strongly agree to strongly disagree for questions related to attitude and ranging from very likely to very unlikely for questions related to practices. The questionnaire was piloted and no major modifications were made. KHCC-IRB approval was sought before any formal data collection started.

A one-day workshop was held four times at KHCC; two in March and two in July of 2019, in English. Instructors from the SHBPP (T.A) and the University of Tennessee, Memphis (H.M) were hosted. The training included didactic introductory lectures including a lecture on the newly enacted MHLL, by the President of the Medical Association in Jordan. Group exercises were conducted using short and long case scenarios. In the short case scenarios, participants were divided and rehearsed the short cases in pairs, followed by disclosure of the event in front of the attendees, then receiving feedback by the instructors. Two long-case scenarios followed this with role-playing by a professional actor and actress, who were given the headline of the case studies; they then improvised according to the way the medical error was disclosed by the participating physicians. Notes were collected by the instructors during the scenarios and a comprehensive feedback from the instructors and actors as well as participants themselves was shared with the physicians who played the role. This was a convenient sample. At the end of the day, physicians who attended the training (*n* = 55) were invited to complete the questionnaire. Physicians who did not attend the training (*n* = 33) were approached at the end of the formal training sessions during August and September in 2019 and handed a copy to complete the questionnaire. To assure anonymity and transparency of responses, identifiers were not collected, including the names, departments and employee ID number. The training team requested a limited number of participants to facilitate interaction with the trainers with feedback, especially at the conclusion of each of the case scenarios. The organizer (A.M, M. H and A.T) sent invitations to all departments at KHCC including the Department of Medical Oncology, Surgery, Pediatrics, Radiotherapy, Radiology, Nuclear Medicine, and Pathology and Laboratory Medicine, as well as the various programs including the Palliative care, Psychosocial among others. Since the training was repeated several times, consultants with different levels of experience were primarily targeted, especially those in direct contact with patients. In total, there were 22 physicians’ participants/ workshop out of 133 (41.4%) physicians at KHCC.

Data was analyzed with SPSS 19 software. Descriptive statistics were used to present the demographics and KAP. Likert-scale responses were dichotomized as follows: (1) likely/very likely versus not sure/unlikely/very unlikely and (2) agree/strongly agree versus neutral/disagree/strongly disagree. To simplify reporting in the “Results” section, likely signifies the combination of “likely” and “very likely” responses, and agree signifies the combination of “agree” and “strongly agree” responses. Missing data was minimal (< 1.0%) and was excluded from the analysis.

The differences between response(s) of physicians’ who received the training and who did not were analyzed with chi-square test and ANOVA to find out the correlation, with a *p*-value of 0.05 used as a cutoff measure for statistical significance.

## Results

The questionnaire was completed by 88 physicians working at KHCC; 55 (62.50%) received training at the workshop, and 33 (37.50%) did not. There were 57 (64.8%) males and 30 (34.1%) females with a male to female ratio of 1.9:1. There was no significant difference among the distribution of gender in both groups. The mean medical practice experience of all physicians was 16.5 years (range: 2–40 years). The mean experience for those who attended the workshop was 17.5 years, compared to 14.8 years of those who didn’t (*p*-value = 0.169). Table 1 shows the participants’ characteristics.

**Table 1:** Demographics of the study cohort

		Received Training ***N*** = 55	Did not received training ***N*** = 33
**Gender**	Male	34 (61.8%)	23 (69.7%)
	Female	20 (36.4%)	10 (30.3%)
**Experience (Years)**	Min	3	2
	Max	40	32
	Mean	17.54	14.82

No difference in medical error disclosure between participants in terms of gender and years of experience were found, whether they attended the workshop or not (Table 2).

**Table 2:** Comparison of gender and years of experience in the total cohort

Variable			Agree	Disagree	***P***-value
**Gender**	Male	Count (%)	39 (68.4%)	18 (31.6%)	0.786
	Female	Count (%)	19 (65.5%)	10 (34.5%)	
**Experience**		Count (median years of experience)	56 (15.84)	28 (17.82)	0.340

We asked physicians about their likelihood of disclosure of errors that caused no harm, minor harm, and major harm. Eighty seven percent (*n* = 73) of respondents (both groups) were more likely to report major harm, compared to minor harm or no harm (*p*-value 0.000). Table 3 displays the relation between the level of harm and the percentage of reporting physicians across all physicians, while Table 4 compares the results between those who attended and those who did not.

**Table 3:** Displays the relation between the level of harm and the percentage of physicians likely to disclose them

Harm Caused	Number (%) of respondents who were likely to disclose	***P*** value
**No Harm**	49 (57.6%)	0.000
**Minor Harm**	67 (76.1%)	
**Major Harm**	73 (86.9%)

**Table 4:** Major harm was the level at which physicians would disclose to patients

Harm Caused	Received Training	Number (%) of respondents who were likely to disclosure	***P***-value
**No Harm**	Yes	26 (50.0)	0.179
	No	23 (69.7)	
**Minor Harm**	Yes	40 (76.9)	0.966
	No	27 (81.8)	
**Major Harm**	Yes	47 (92.2)	0.162
	No	26 (78.8)	

Jordan’s Law on Medical and Healthcare Liability was addressed in two questions especially that this law came into effect recently. Physicians who attended the workshop were more knowledgeable of its' articles and elements than those who did not attend (*p*-value = 0.017). More so, the difference in knowledge on decision of error disclosure was in favor of physicians who have attended the workshop (*p*-value = 0.024). The majority of physicians in both groups preferred disclosure of medical errors because it is how they would want to be treated if they were patients (96.4 and 87.8%, respectively, *p*-value = 0.103). They also believed that disclosure of medical errors would help them alleviate the feeling of guilt (66.7 and 45.5%, respectively, *p*-value = 0.121). Almost half the physicians in both groups believed that disclosure of medical error would not strengthen the doctor-patient relationship (50.9 and 42.4%, respectively, *p*-value = 0.24). Interestingly though, physicians who received training were more inclined to disclose errors depending on their perception of whether this will help or harm the patients (72.7 and 57.6%, respectively, *p*-value =0.032). Table 5 summarizes the attitudes of physicians towards disclosing errors.

**Table 5:** Attitudes toward error disclosure

Questions	Received Training	Number (%) of respondents who agreed	***P***-value
**I am aware and knowledgeable of the articles/elements of Jordan’s Medical and Health Liability Law**	**Yes**	**36 (66.7)**	**0.017**
	**No**	**15 (45.5)**	
**How likely would the issue of Jordan’s Law on Medical and Health Liability affect your decision to disclose an error to your patient?**	**Yes**	**34 (61.8)**	**0.024**
	**No**	**12 (36.4)**	
**It is important for me to tell my patients about errors I have made in their care because that is how I would want to be treated if I were a patient.**	**Yes**	**53 (96.4)**	**0.103**
	**No**	**29 (87.8)**	
**If I made a medical error, disclosing the error to my patient would help alleviate my feelings of guilt.**	**Yes**	**47 (87.0)**	**0.121**
	**No**	**23 (69.7)**	
**Telling my patient about a medical error I have made in their care strengthens my patient’s trust in me as a physician.**	**Yes**	**28 (50.9)**	**0.24**
	**No**	**14 (42.4)**	
**My decision to disclose a medical error to a patient depends on whether I think the information will help or harm him/her.**	**Yes**	**40 (72.7)**	**0.032**
	**No**	**19 (57.6)**	

With respect to professional relationships between physicians and their peers, physicians in both groups felt the need to share the burden of a medical error (76.4% vs 62.5%, *p*-value = 0.24). Physicians in both groups recognized at least one colleague who would give them support if needed (94.5% vs 78.8%, *p*-value = 0.064). Reasons for wanting to share the incident with colleagues included learning whether they would have made the same clinical judgments and decisions (90.9% vs. 78.8%, respectively, *p*-value = 0.149), learning from their errors (85.5% vs. 75.8%, respectively, *p*-value =0.256), getting support and understanding (89.1% vs. 75.8%, respectively, *p*-value = 0.277), strengthening their professional relationships with the team (63.6% vs. 48.5%, respectively, p-value =0.350), and unburdening themselves (67.3% vs. 51.5%, respectively, *p*-value = 0.280). Interestingly, those who witnessed their mentors disclosing errors to patients were more likely to do the same in front of their students and residents than those who did not recall a similar experience with their mentors (71.0% vs. 29.0%, respectively, *p*-value =0.008), which reveals the importance of role modeling and the hidden curriculum.

When it comes to the relation with one’s institution, both groups believed that reporting medical errors to one’s own institution improves the quality of care for future patients (96.3% in the group who received the training and 84.8% who did not, *p*-value = 0.15) and they believed that the benefits of reporting medical errors outweigh the negative consequences for those who report them (67.3% vs 66.7%, respectively, *p*-value = 0.290). However, 85.5% of physicians who received the training knew how to report medical errors to the institution, compared to 66.7% physicians who did not (*p*-value = 0.02). In addition, receiving a feedback from the institution would enhance reporting of medical errors as perceived by those who received training against who did not (85.2% vs 56.3%, *p*-value =0.003).

When thinking about disclosing medical errors, physicians who received the training and physicians who did not were both concerned about malpractice litigation (58.2% vs 59.4%, respectively, *p*-value = 0.042), blame from colleagues (60.0% vs 50.0%, respectively, *p*-value = 0.584), professional discipline (59.3% vs. 67.7%, respectively, *p*-value = 0.720), loss of reputation, (61.8% vs. 56.3%, respectively, *p*-value = 0.842), negative reaction from the patients or their families (58.2% vs 75.6%, respectively, * p*-value = 0.089), and negative publicity in the news media (58.2% vs 65.6%, respectively, *p*-value = 0.564).

## Discussion

The purpose of this study was to investigate the different aspects associated with medical error disclosure among physicians at our center. It also aimed to measure their knowledge of the new MHLL in Jordan and its impact on physicians’ medical error disclosure. To our knowledge, this is the first study that looked into the effect of formal training of physicians on disclosing medical errors after the enactment of the MHLL in 2018. A literature review revealed that only a couple of studies addressing the issue in Jordan were previously published. Our study is different as it measures the KAP among physicians with and without formal training and after the enactment of the MHLL [34, 35]. Notable among our findings is the lack of significant difference in reporting major harm between both groups of physicians (*p*-value = 0.07), physicians who attended the workshop gained better knowledge of the MHLL and the Center’s policies (*p*-value = 0.017), and that these differences in knowledge was reflected on the perceived practice to disclose medical error in physicians who received the training (*p*- value = 0.024). This confidence about the practice of medical error disclosure in physicians who attended the workshop can either be attributed to their knowledge of the MHLL which was addressed during the workshop or to their more comprehensive understanding of the importance of disclosure more after the attendance of the workshop (90.9% vs 66.7%, respectively, *p*-value =0.003). Moreover, 85.5% of physicians who received the training reported better knowledge on how to report medical errors to the institution, compared to 66.7% of physicians who did not attend (*p*-value = 0.02). This comes in alignment with published literature on the effectiveness of educational workshops in enhancing medical error disclosure [36, 37]. When medical error disclosure KAP was stratified according to gender and years of experience, our study found no difference between respondents in terms of gender and experience whether they attended the workshop or not. While some studies showed no significant gender differences regarding medical error disclosure, reporting was affected by years of experience, and physicians with more years of experience were more likely to report medical errors resulting in no harm, minor harm, and major harm than residents and physicians with less years of experience [33]. This variation in findings can be explained by the sample size in our study and the homogeneity of our participants.

With respect to physician-to-physician communication and collegial relationships, physicians in both groups were able to recognize at least one colleague who would support them when needed (*p*-value = 0.064). They identified many reasons on why they need to share medical errors such as clarification and reassurance, to see what clinical judgment their peers would have made, and to receive the much needed emotional and moral support and understanding. This emphasizes the under-recognized need for psychological and moral support of physicians [14], which should be perceived as a learning opportunity for physicians and institutions as the toll of an medical error can be heaving on physicians themselves and thus, they too, in a way, are a victim of medical errors.

Research showed that the presence of role models and mentors to medical students or physicians has a key impact on improving the competency in medical error disclosure through providing a passive, experiential method of learning for these individuals [38, 39]. In our study, physicians who had witnessed a past experience of their mentors disclosing errors to patients were more likely to do it themselves than those who did not recall a similar experience with their mentors (*p*-value = 0.008), which highlights the important role senior/ experienced physicians plays in shaping the future practice of residents and junior physicians and how they can significantly improve their experience and capabilities of medical error disclosure. A potential venue for discussing and teaching medical errors and the significance of disclosure is through incorporating these in the curriculum of medical schools or residency programs, which to our knowledge, current curriculums are lacking. This will also ensure that, later on, these physicians will be role models to others.

As to the physicians’ relation with the institution, more physicians attending the workshop knew how to report medical errors to the institution, compared to physicians who did not attend (*p*-value = 0.02). Furthermore, physicians who attended the workshop were more inclined to report medical errors given that they would receive feedback from the institution (*p*-value =0.003). Thus, orientation of newly hired physicians and a continuous update to physicians at the center might be a prerequisite for the continued professional development scheme. Moreover, building a no shame- no blame culture in the institution is very much warranted and will provide a safe space for physicians to enhance a culture of transparency.

Medical error disclosure can be more sensitive in cancer care, an environment where medical errors can be especially detrimental. In literature, oncologists agreed that medical errors should be disclosed. However, fear of legal litigation was one of the main barriers to error disclosure, as well as fear of negative reaction or distrust from their patients, and institutional pressure regarding medical error disclosure [40, 41], which is concordant with our findings at KHCC. One other aspect is that oncologists tend to carry more emotional burden when medical errors occur to their patients [27], further stressing the need for emotional and psychological support to physicians from their peers and their institution when faced with medical errors.

We acknowledge some limitations in our study. First, the data was obtained from a single center with a relatively small number of participants. In addition, while the official language in Jordan is Arabic, all workshops and the role-playing were held in English, a factor that does not mirror real-world practice where medical errors have to be disclosed in Arabic. Language was a barrier to some of the attendees. Indeed, when the trainers suggested that one simulation be done in Arabic, even the way the error was disclosed was more effective. This would call to assimilate some workshops in Arabic. Likewise, future studies, tailored to accommodate the cultural norms in each community, are in need; whereas the patient and one to a few relatives are involved in disclosure of errors, the need to disclose and explain the encounter is expected to involve many individuals including the extended families. Also, although physicians working at KHCC were the primary target, and despite the small sample size, the results can probably be generalized to physicians in other institutions and specialties since the topics discussed, the update on the new MHLL, and the case scenarios were not cancer specific. However, repeating the training to include more physicians from other sectors is warranted.

## Conclusions

Education of physicians on error disclosure by conducting the workshops using simulation helped physicians become more knowledgeable and competent about on this vital issue. The workshop improved their comprehension on the MHLL, as the attendees declared it as the one of the most important factors in wanting to learn how to disclose medical errors. We also acknowledge the support needed from peers and institutions, as well as the importance of a professional role model. We recommend extending the workshops to more institutions, and to incorporate it in medical and training curricula at different universities. We also recommend adding training on medical error disclosure and the local policies per institution, as well as raising more awareness of the MHLL. Further studies are recommended where other institutions and more physicians are involved. The value of integrating such training in the curricula of medical schools and residency training programs cannot be over-emphasized.

## Supplementary information

**Additional file 1.**

## Data Availability

The dataset supporting the conclusions of this article is available with the Corresponding author and is present upon request.

## Abbreviations

AUBMC: American University of Beirut Faculty of Medicine and Medical Center
HEC: Hospital Ethics Committee
IRB: The Institutional Review Board
KAP: Knowledge, attitudes and practices
KHCC: King Hussein Cancer Center
MHLL: Medical and Health Liability Law
SHBPP: Salim El-Hoss Bioethics and Professionalism Program
