# Supplementary material for: Disclosure of medical errors: physicians’ knowledge, attitudes and practices (KAP) in an oncology center
Source: BMC Med Ethics. 2020 Aug 20;21:74. doi: 10.1186/s12910-020-00513-2 (PMC7439528; doi:10.1186/s12910-020-00513-2)
Supplement: Supplementary file 1 — Additional file 1. [file 12910_2020_513_MOESM1_ESM.docx]

Dear colleagues,

The research team invites you to complete this research survey entitled “**Disclosure of Medical Errors: Physcians’ Practices and Attitudes in an Oncology Center**”. This research survey is for the purpose of learning more about physcians’ practices and attitudes towards disclousre of medical errors at our center and how training initiatives can influence such practices and attitudes.

Your participation is **voluntary**, you may chose not to complete the survey or stop at any time. The survey is **anonymous** and your responses will not affect your employment in any way. It does not pose any risk to you other than the inconvenience in taking the time to complete the survey. The survey will take no more than **5-10 minutes** to complete. The information that you provide will remain strictly **confidential**.Your information will be combined with other responses.

If you have any further questions, please don't hesitate to contact Dr. Maysa Al-Hussaini on 065300460/ **Ext**.1308 or by email [mhussaini@KHCC.JO](mailto:mhussaini@KHCC.JO)

This study was reviewed and approved by KHCC-IRB. If you have any questions or concerns related to your right as a research participants, please contact the IRB Office on 065300460/ Ext.1669 or by email [irboffice@KHCC.JO](mailto:irboffice@KHCC.JO)

**Thank you for your kind cooperation and time. We highly value your views.**

| **Demographics** |
| --- |
| **Gender:** ❑male ❑ female |
| **Total years of experience: ____________** |

| **Communicating with Patients** | |
| --- | --- |
| How likely would you disclose to your patient an error that caused:   1. No harm? 2. Minor harm? 3. Major harm? | Very Likely Not Sure Unlikely Very  Likely Unlikely  ❑❑❑❑ ❑  ❑❑❑❑ ❑  ❑❑❑❑ ❑ |
| When a medical error occurs, I feel an obligation:   1. to tell my patient the facts necessary forhim/her to understandwhat happened 2. to make it clear that what happened wasaerror. | Strongly Strongly  Agree Agree Neutral Disagree Disagree  ❑ ❑ ❑ ❑❑  ❑ ❑ ❑ ❑❑ |
| Disclosing medical errors to patients is the right thing to do evenif it comes at a significant personal cost (e.g., harms my reputation or increases my malpractice risk). | Strongly Strongly  Agree Agree Neutral Disagree Disagree  ❑ ❑ ❑ ❑ ❑ |
| It is important for me to tell my patients about errors I have made in their care because that is how I would want to be treated if I were a patient. | Strongly Strongly  Agree Agree Neutral Disagree Disagree  ❑ ❑ ❑ ❑ ❑ |
| If I made a medical error, disclosing the error to my patient would help alleviate my feelings of guilt. | Strongly Strongly  Agree Agree Neutral Disagree Disagree  ❑ ❑ ❑ ❑ ❑ |
| Telling my patient about a medical error I have made in their care strengthens my patient’s trust in me as a physician. | Strongly Strongly  Agree Agree Neutral Disagree Disagree  ❑ ❑ ❑ ❑ ❑ |
| My decision to disclose a medical error to a patient depends on whether I think the information will help or harm him/her. | Strongly Strongly  Agree Agree Neutral Disagree Disagree  ❑ ❑ ❑ ❑ ❑ |
| I am aware and knowledgeable of the articles/elements of Jordan’s Law on Medical and Health Liability/ قانونالمسؤوليةالطبيةوالصحية. | Strongly Strongly  Agree Agree Neutral Disagree Disagree  ❑❑❑❑ ❑ |
| How likely would the issue of Jordan’s Law on Medical and Health Liability/قانونالمسؤوليةالطبيةوالصحية,affect your decision to disclose an error to your patient? | Very Likely Not Sure Unlikely Very  Likely Unlikely  ❑❑❑❑ ❑ |

| **Professional Relationships** | |
| --- | --- |
| I usually discuss my medical errors with colleagues. | Strongly Strongly  Agree Agree Neutral Disagree Disagree  ❑ ❑ ❑ ❑ ❑ |
| I know at least one colleague who would support me if I needed to talk with him/her about a medical error I made. | Strongly Strongly  Agree Agree Neutral Disagree Disagree  ❑ ❑ ❑ ❑ ❑ |
| The following reasons motivate me to discuss my medical  errors withmycolleagues: Strongly Strongly Agree Agree Neutral Disagree Disagree   1. To learn whether they would have madethe   same clinical judgments and decisionsIdid ❑ ❑ ❑ ❑ ❑   1. To allow them to learn frommy errors ❑ ❑ ❑ ❑ ❑ 2. To receive their supportandunderstanding❑ ❑ ❑ ❑ ❑ 3. To strengthens myprofessional relationships ❑ ❑ ❑ ❑ ❑   with them   1. Tounburden myself ❑ ❑ ❑ ❑ ❑ | |
| When I was a student or resident, I was able to observe (at least once), a more experienced clinician discuss his or her medical error with me or another clinician. | ❑Yes ❑No ❑ Unable toremember |
| I have tried (at least once) to serve as a role model to students or residents by discussing one of my own medical errors with them. | ❑Yes ❑No ❑ Unable toremember |

| **Reporting Errors** | |
| --- | --- |
| Reporting medical errors to one’s own institution improves the quality of care for future patients. | Strongly Strongly  Agree Agree Neutral Disagree Disagree  ❑ ❑ ❑ ❑ ❑ |
| I know how to report medical errors to my institution. | Strongly Strongly  Agree Agree Neutral Disagree Disagree  ❑ ❑ ❑ ❑ ❑ |
| I know what kinds of medical errors should be reported to my institution. | Strongly Strongly  Agree Agree Neutral Disagree Disagree  ❑ ❑ ❑ ❑ ❑ |
| It is hard to be certain about the true causes of adverse events in the practice of clinical medicine. | Strongly Strongly  Agree Agree Neutral Disagree Disagree  ❑ ❑ ❑ ❑ ❑ |
| I would be more likely to report errors to my institution if I knew I would receive feedback afterwards. | Strongly Strongly  Agree Agree Neutral Disagree Disagree  ❑ ❑ ❑ ❑ ❑ |
| Disclosing errors to my institution isn’t worth my time because my actions can’t change the system of care. | Strongly Strongly  Agree Agree Neutral Disagree Disagree  ❑ ❑ ❑ ❑ ❑ |
| Overall, I believe the benefits of reporting medical errors are outweighed by the negative consequences for those who report them. | Strongly Strongly  Agree Agree Neutral Disagree Disagree  ❑ ❑ ❑ ❑ ❑ |

| **Concerns about Disclosing Errors** |
| --- |
| In general, when thinking about disclosing medical errors,  I am concerned about the followingpossibleconsequences: Strongly Strongly  Agree Agree Neutral Disagree Disagree   1. Blame fromcolleagues ❑ ❑ ❑ ❑ ❑ 2. Disciplinary action by aprofessionalbody ❑ ❑ ❑ ❑ ❑ 3. Loss of reputationamongcolleagues ❑ ❑ ❑ ❑ ❑ 4. Malpracticelitigation ❑ ❑ ❑ ❑ ❑ 5. Negative patient orfamily reaction ❑ ❑ ❑ ❑ ❑ 6. Negative publicity fromnews media ❑ ❑ ❑ ❑ ❑ |

**Any additional comments you would like to share with the research team:**

**______________________________________________________________________________________________________________________________________________________________________________________________________________________________________________________________________________________________________**

Thank You!
